# Speaker Input Variability Does Not Explain Why Larger Populations Have Simpler Languages

**DOI:** 10.1371/journal.pone.0129463

**Published:** 2015-06-09

**Authors:** Mark Atkinson, Simon Kirby, Kenny Smith

**Affiliations:** Language Evolution and Computation Research Unit, School of Philosophy, Psychology and Language Sciences, University of Edinburgh, Edinburgh, United Kingdom; University of Stirling, UNITED KINGDOM

## Abstract

A learner’s linguistic input is more variable if it comes from a greater number of speakers. Higher *speaker input variability* has been shown to facilitate the acquisition of phonemic boundaries, since data drawn from multiple speakers provides more information about the distribution of phonemes in a speech community. It has also been proposed that speaker input variability may have a systematic influence on individual-level learning of morphology, which can in turn influence the group-level characteristics of a language. Languages spoken by larger groups of people have less complex morphology than those spoken in smaller communities. While a mechanism by which the number of speakers could have such an effect is yet to be convincingly identified, differences in speaker input variability, which is thought to be larger in larger groups, may provide an explanation. By hindering the acquisition, and hence faithful cross-generational transfer, of complex morphology, higher speaker input variability may result in structural simplification. We assess this claim in two experiments which investigate the effect of such variability on language learning, considering its influence on a learner’s ability to segment a continuous speech stream and acquire a morphologically complex miniature language. We ultimately find no evidence to support the proposal that speaker input variability influences language learning and so cannot support the hypothesis that it explains how population size determines the structural properties of language.

## Introduction

Languages evolve, adapting to pressures which arise from their learning and use [1]. As these pressures may be different in different physical, demographic and sociocultural environments, non-linguistic factors may systematically determine linguistic features [2–5]. Identifying those factors which specifically affect the structural properties of language, and establishing the mechanisms by which they operate, will shed light on why languages exhibit different degrees of grammatical complexity [4] and how individual-level learning interacts with the sociocultural features of a speech community to result in group-level language features [6–8]. It may also aid our understanding of typological and psycholinguistic constraints on language [2–4], as well as provide clues as to the emergence of structure in the early language of our species [2].

At the level of the individual learner, the language an individual acquires depends on the specific linguistic input they receive, the context in which it is transmitted, and the way that input interacts with the learning abilities and biases of the learner [5, 8, 9]. Across different types of groups in different environments, there may be systematic differences in the input data learners receive and the effect it has on their developing languages. This may explain observable differences in languages spoken by different types of social groups in different environments [2–5, 10].

Here we consider one particular feature of the linguistic input, the degree of homogeneity in the data arising from the number of speakers who provide it. It has been suggested that this difference may have systematic effects on the acquisition of complex morphology, and that this may result in the simplified morphological systems seen in the languages of larger groups [5].

### Speaker input variability and phoneme acquisition

Variability in linguistic input can arise at multiple levels of analysis, from different lexical items or word orders being used to convey the same semantic information down to subtle variability in the realisation of phonemes. One source of the latter kind of variability is the differences in the idiosyncratic pronunciations of the speakers who provide the input. This results from dialectal differences and variable speech rates, as well as anatomical differences amongst the speakers, such as the length and shapes of their oral and nasal cavities [11, 12]. *Speaker input variability* may therefore be increased either by the pronunciation being less homogeneous across the speakers, or by the data being provided by a greater number of speakers [5].

A number of studies have demonstrated the effect that input variability can have on the acquisition of phonemic (or tonal [13] contrasts. These studies consider adult second language acquisition and typically focus on Japanese learners of English attempting to acquire the contrast between /l/ and /r/. Input variability is manipulated by either exposing learners to target phonemic distinctions in a greater number of lexical contexts, or by considering the effect of High Variability Phonetic Training (HVPT), where the learner is simply exposed to “natural words from multiple talkers” [14, p. 3267]. Both types of variability aid discrimination of target phonemic contrasts [12, 15–17], with a direct comparison of the two manipulations finding HVPT more effective than context variability [18]. The effects of HVPT have also been confirmed in discrimination tasks involving familiar and novel speakers [15, 16, 18], for retention of phonemic boundaries 6 months after training [15], and in learner productions [16, 19].

This evidence that increasing speaker input variability can aid phoneme acquisition, and by extension minimal pairs of a lexical set, is alone enough to suggest that its effect on other aspects of language acquisition is worth investigation. But it has also been proposed that speaker input variability may explain how non-linguistic features of a speech community could influence structural features of its language.

### Sociocultural determination of linguistic structure

A body of work has already aimed to identify the sociocultural factors which influence non-structural features of language. For example, the number, specificity and semantic complexity of lexical items results from a group’s need for and ability to maintain distinctions [2, 3, 20]: distinguishing amongst different types of sheep will be more useful to sheep farmers than other social groups, and so the lexicon of a British sheep farmer will include terms such as *gimmer*, *freemartin* and *rigger*, which may well be unfamiliar terms to other speakers [3]. Perhaps more speculatively, phoneme inventories and phonotactic constraints are thought to have adapted to have a greater proportion of more sonorant phonemes in environments which favour more distal communication, such as in warmer climates or where there is less vegetation [21–23]. The size of a language’s phoneme inventory may also be influenced by its number of speakers: languages of larger groups have been claimed to have larger phoneme sets [24–26].

There is a growing interest in how demographic or sociocultural factors may determine **structural** features of a language [5]. Wray and Grace [2] discuss how different sizes and types of social group might influence systematic differences in the complexity of their languages, considering two extremes of communication: esoteric, or intra-group, and exoteric, or inter-group, communication. They argue that esoteric communication, as used by speakers in small, unified social contexts where a lot of information can be presupposed, will be more complex. There will be a greater number of irregular and opaque features, a higher degree of morphological complexity with a greater number of irregularities (note that use of morphological strategies over lexical is in itself likely to result in an increase in the number of irregular forms [27]) and more derivational constraints leading to increased suppletion. Conversely, exoteric communication is that employed by larger groups, with a large amount of interaction conducted between strangers and therefore with more limited shared information for interlocutors to rely on. Such communication will be less grammatically complex, characterised by one-to-one relations between form and meaning, allomorphy, regularity, transparency, flexibility of expression and compositionality of signals. Wray and Grace argue that the complex nature of esoteric communication is more representative of the “default” psycholinguistic preference for less regular and transparent language, and so will be the result of languages which prioritise child language learning and the communicative needs of more intimate social groups. Simpler, exoteric, communication is then a “consequence[] of talking to strangers” [2, p. 543], where the language has adapted to the needs of adult language learning. Trudgill [3] also argues that more complex languages are more likely to be found in situations where there is less contact with other languages, higher social stability, smaller speech communities, denser social networks and more “communally-shared information” [3, p. 146].

These claims receive empirical support from work by Lupyan and Dale’s study of the correlation between demography and morphological complexity [10]. Following previous work investigating the relationship between the number of speakers of a language and grammatical complexity [28, 29], they investigate 2,236 languages using data from the World Atlas of Language Structures database [30], considering 28 structural features relating to each language’s morphological type, case system, verb morphology, agreement, possibility and evidentials, negation, plurality, interrogatives, tense, possession, aspect, mood, articles, demonstratives and pronouns. Controlling for language family and geographic location, they find that languages with larger populations, spoken over larger areas and in contact with a greater number of other languages tend to be characterised by lower morphological complexity and the greater use of lexical strategies to make semantic distinctions. They found that population size had the most predictive power, and specifically claim that languages spoken by a greater number of people have less complex inflectional morphology. More recently, simulations of language learning have also supported the proposal that the languages of larger groups are likely to have a greater number of simpler conventions which are easier for a learner to acquire [31].

### Speaker input variability and structural complexity

Discovering a correlation between a non-linguistic factor such as number of speakers and the structural features of a language is not satisfactory in itself: a causal mechanism needs to be identified to explain why and how a proposed determinant could have such an effect. Population size itself may actually not be the most informative predictor. There may instead be a more direct determinant, some aspect of society or environment which is itself correlated with larger groups [5]. Alternatively, the effect may be the result of the interaction of a number of factors [3], with features, such as cultural complexity [32, 33], whether or not the language has a written form [2, 34] and language age [35], also having some influence.

One proposed explanation, discussed by Nettle [5], is the differing degrees of speaker input variability encountered by learners in different sized groups. Nettle suggests that an individual’s social network will be more constrained in smaller populations. The input they receive is therefore likely to be more homogeneous, being provided by a smaller number of speakers, or otherwise exhibiting less inter-speaker variability due to the reduced possibilities for dialectal differences. In larger groups, the learner is part of a larger social network, and so the input they receive is likely to be more variable. Nettle proposes that increased variability makes morphological distinctions, which are often based on minimal phonological differences, more difficult to acquire and hence less likely to survive cross-generational transfer. With the loss of these comparatively subtle distinctions, an alternative strategy is necessary if the same semantic distinctions are to be maintained. This is likely to be an innovated, structurally more simple, lexical strategy [5].

A challenge for this proposal is to explain why greater input variability aids phoneme acquisition yet hampers the acquisition of morphology [5]. One solution is to note the very different roles that increased variability may have in each case. In the acquisition of a phoneme set, higher variability provides more information about the group-level distribution of a phoneme and so aids the maintenance of phonemic distinctions. In the acquisition of morphology, however, it may simply increase the noise in the input and make the target less accessible to the learner. Such an account may explain why languages of larger groups appear to have both larger phoneme sets [24–26] (though see [36]) and simpler morphological systems [5, 10].

In the remainder of this paper we describe two experiments designed to test the effects of speaker input variability on language acquisition, and therefore test the plausibility of speaker input variability as a mechanism explaining how group size influences morphological complexity. In Experiment 1, we extended previous work on statistical learning to consider whether the effect of speaker variability in phoneme acquisition can be extended to word segmentation. In Experiment 2, we tested the effect of speaker input variability on the learning of a morphological system. To anticipate our results: we find no evidence that increased speaker input variability impedes (or indeed facilitates) the learning of morphology, therefore throwing some doubt on the viability of this mechanism.

## Experiment 1: word segmentation

In their seminal study investigating the abilities of learners to use distributional cues to segment continuous linguistic input, Saffran et al. [37] demonstrated that adults were able to segment words from a speech stream using only the transitional probabilities between consonant-vowel (CV) syllables. These abilities have since been extended to infants [38], natural speech [39], larger learning sets [40], the acquisition of multiple languages [41], non-linguistic auditory tasks [42], equivalent capabilities in the visual field [43, 44] and even to other species [45].

The transitional probability between the elements of an input stream is computed by dividing the frequency of a pair of units *XY* by the frequency of the unit *X*. A higher probability then indicates that the presence of element *X* more strongly predicts the subsequent presence of *Y*. An example, taken from Saffran et al. [37, p. 610], considers the syllable as the unit of analysis and the English word *baby* (/beɪ.bi/). /beɪ/ is a relatively high-frequency syllable, which will be followed by /bi/ some of the time. But it can also be followed by other syllables, both within a word, as in *bacon* or *baker*, or across a word boundary, as in *Bay of* or *obey the*. Since words can be freely combined (within the syntactic constraints of a language), the predictability of a second element in a pair of syllables within words will generally be higher than those which span a word boundary, and so the probability of, for example, /bi/ following /bei/ is likely to be higher than /ðə/ following /bei/. Therefore the transitional probability of /beɪ.bi/ would then be higher than /bei#ðə/. Transitional probabilities therefore form a cue which can be used to identify the components of an input stream: while in the statistical learning literature these are typically glossed as words, the same logic applies to the segmentation of complex signals built by productive morphological processes.

Determining the morpheme boundaries of input data is one of the first steps in the acquisition of a morphological system [37, 40]. Therefore if increased speaker input variability makes the segmentation of a speech stream more difficult, a learner may find the acquisition of complex morphology more challenging; this may eventually result in the language simplifying as it is transmitted from learner to learner [5]. To assess this, we adapted the experimental design of Saffran et al. [37] to investigate whether or not there is an effect of the number of speakers who provide the input. To our knowledge, this is the first investigation of the effect of speaker input variability on word segmentation and the first attempt to see if the findings of the HVPT studies can be extended to other aspects of language acquisition.

## Materials and methods

This experiment was approved by the Linguistics and English Language Ethics Committee of the University of Edinburgh. Written consent was provided by all participants before taking part.

The methodology for this experiment was based on the first experiment described in Saffran et al. [37], with an additional manipulation of speaker input variability. We assessed the ability of adult learners to discriminate between words and non-words in forced-choice testing after exposure to a continuous speech stream. In the single speaker condition, the learner’s input came from a single speaker; in the multiple speaker condition, the input was instead spread among 3 different speakers.

Following Saffran et al. [37], four consonants (p, t, b, d) and three vowels (a, i, u) were used to construct an inventory of 12 CV syllables, from which six trisyllabic words were created (*babupu*, *bupada*, *dutaba*, *patubi*, *pidabu*, *tutibu*). An aural stimulus was constructed by concatenating the words of the language into a continuous speech stream, lacking acoustic cues to word boundaries. 300 tokens of each word were randomly ordered, with words then eliminated so that no adjacent words were the same. In contrast to Saffran et al. [37], and to reduce any influence of the order of a particular input string, we generated 24 such input strings, each independently randomised, and used each once only in each experimental condition. In each string, the transitional probabilities within a word were greater than the transitional probabilities across a word boundary, as in the original study. For each of the 24 input strings, 6 trisyllablic non-word foils were randomly constructed using the 12 syllables of the CV inventory, but with the stipulation that the transitional probabilities between the syllables within the speech stream was 0. One foil set, for example, was *bubidi*, *tabidi*, *tatupa*, *dubati*, *bitapi* and *tupati*.

As in previous studies [40, 46], the target words, input streams and foils were created using the MBROLA speech synthesis package [47], with a CV syllable duration of 278ms [37], of which 60ms was assigned to the consonant. 4 diphone databases were used to construct each target, input stream and foil for each of 4 different speakers. A constant F0 of 100Hz was assigned to 3 male voices (en1, us2, and de1) [40, 46], and 200Hz for 1 female voice (us1) [48]. Use of synthesised speech ensured that there were no acoustic cues to word boundaries.

### Participants

48 native English speakers (10 male; aged between 18 and 33, mean 21.1) were recruited using the Student and Graduate Employment (SAGE) database of the Careers Service of the University of Edinburgh. Each was compensated £5.50.

### Procedure

As in Saffran et al. [37], the participants were told they were going to listen to a “nonsense” language, which contained words, but no meanings or grammar. They were told “Your task is to try and figure out where the words begin and end. You don’t know how many words there are, nor how long they might be”. To justify the unnaturalness of the monotone stimuli, the language was described as a “robot” language, with the speakers being native robot speakers of the language. Though explicit instruction may influence learning [49–52] (though see [53]), it was not anticipated that replicating the previous study’s instructions [37] would negatively affect the participants’ ability to identify the word boundaries.

**Fig 1:**
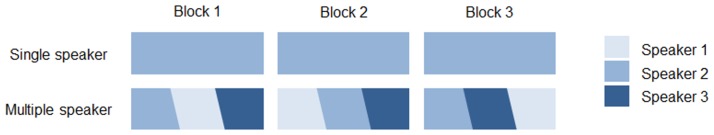
Example training regimes for participants in the single and multiple speaker conditions.

Following Saffran et al. [37], the training strings were split into 3 blocks of approximately 7 minutes each, presented with a 5 minute rest after the first and second blocks. In the single-speaker condition (24 participants), a participant was trained using a single voice, with the voice used counterbalanced across participants (6 participants being trained by each of the 4 voices). In the multiple-speaker condition (24 participants), a participant was trained using 3 of the 4 different voices, with the voices used counterbalanced across participants (6 participants being trained by each of the 4 possible combinations of 3 voices). In this multiple speaker condition, each of the training voices provided a third of the input in each of the 3 blocks in a random order. The multiple-voice audio files were created using Audacity 2.0.5, with 5 seconds of cross-fade between speakers, so as not to provide any additional cues as to the word boundaries at the changeover points. The difference between the training regimes in each condition is illustrated in Fig 1.

Training was followed by two forced-choice testing blocks: one with the stimuli presented by the speaker(s) used in training and one using a novel speaker. In each test block, a participant was presented with all 36 possible word-foil pairings, presented in a random order. For each pairing, the word and foil were presented in a random order with 500ms of silence between them. The participant was required to “decide which of the words is from the robot language”. There was then a 2 second pause before the next pairing.

The familiar-voice block was designed to replicate Saffran et al. [37], while the novel-voice test was included to investigate any possible effect of multiple-speaker training and the comprehension of an unfamiliar speaker, following similar findings in HVPT [15, 16, 18]. To control for any ordering effects, the blocks were counterbalanced so that half the participants in each condition were presented with the familiar speaker test first and half with the novel speaker test first.

For a participant in the single-speaker condition, the familiar-voice testing block used the same voice as in training. The novel-voice block used one of the other 3 voices. Over the set of single-speaker participants, each combination of familiar voice and novel voice was used twice. For a participant in the multiple-speaker condition, each of the voices from the training were used for a third of the testing pairings in the familiar-voice block. The novel-voice block then used the only voice not used in training.

The experiment was written and run in Matlab (R2013b) with the Psychtoolbox extensions.

## Analysis and results

**Fig 2:**
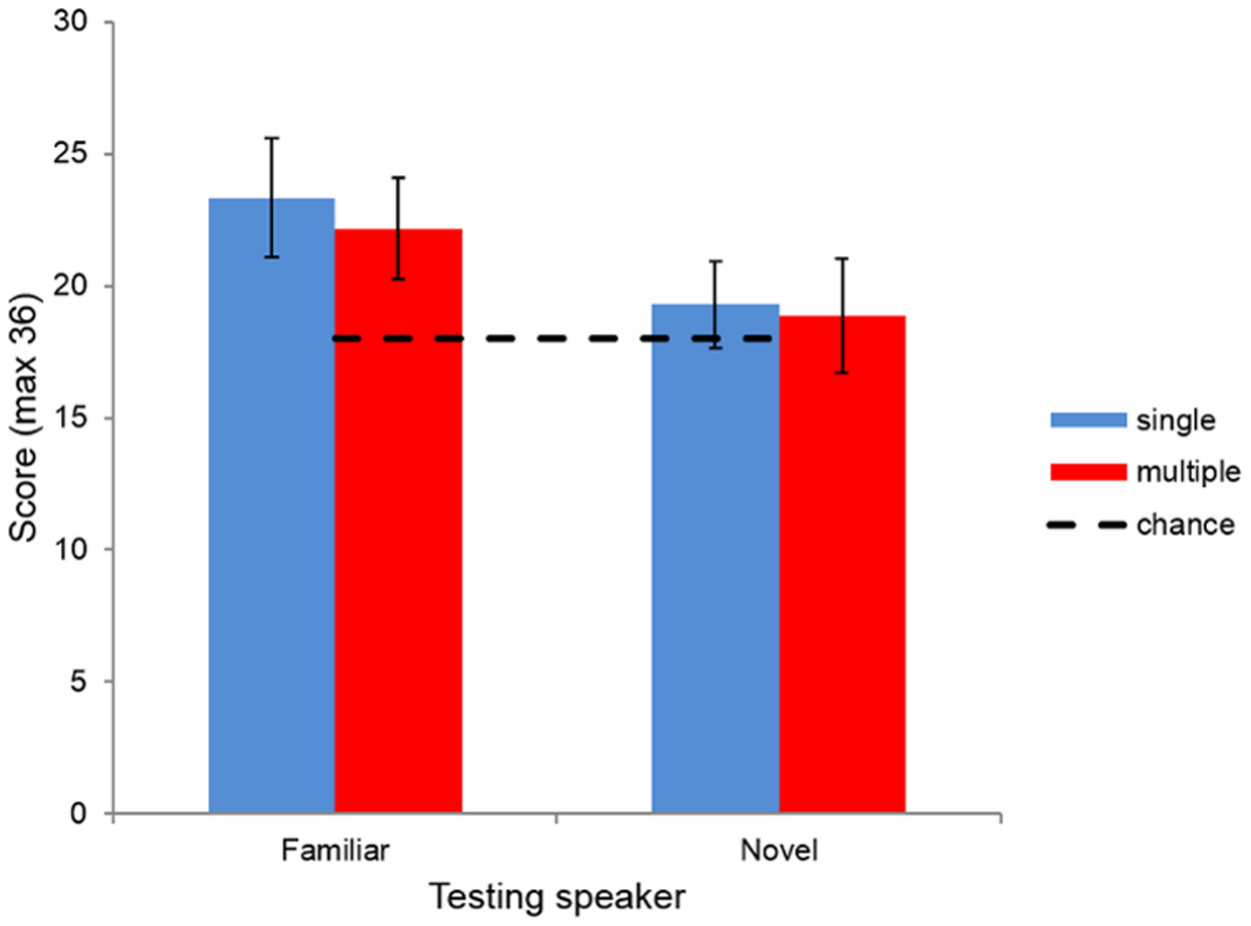
Average scores for each condition in both familiar speaker and novel speaker testing blocks. Error bars represent 95% confidence intervals of the mean in each case.

Learning was assessed by counting the number of times the word was correctly identified in the word-foil test pairings. The maximum score in each block was 36, with chance performance 18. The results are shown in Fig 2.

We performed a linear mixed effects analysis using R [54] and *lme4* [55]. We fit a maximal model [56] with logit regression including *condition* (single speaker or multiple speaker), *speaker identity* (familiar or novel), *order of tests* (familiar speaker test first or second) and the interaction of *condition* and *speaker identity* as (centred) fixed effects, with *participant identity* as a random effect. The interaction of *condition* and *speaker identity* was included to see if there was any effect of participants in the multiple speaker condition being better at distinguishing words from foils when listening to unfamiliar speakers, following similar findings in HVPT [15, 16, 18]. The model was significantly better than the equivalent null model (*χ* ^2^(4) = 52.457, p <0.001). The intercept was significantly different from zero (*β* = 0.343, SE = 0.065, p <0.001), reflecting that, averaging across all our data, participants performed significantly better than chance (participants were 1.41 times as likely to produce a correct response on test as incorrect, corresponding to an accuracy of 58%). There were significant contributions of *speaker identity* (*β* = -0.439, SE = 0.070, p <0.001) and *order of tests* (*β* = -0.258, SE = 0.070, p <0.001). There were no effects of *condition* (*β* = 0.097, SE = 0.130, p = 0.457) or the interaction of *condition* and *speaker identity* (*β* = -0.093, SE = 0.141, p = 0.508).

This analysis suggests that the participants were able to use the distributional cues in their training strings to discriminate between words and non-words, replicating the result of Saffran et al. [37]. Performance was better in the familiar voice testing: participants were 1.76 times as likely to produce a correct response as incorrect, corresponding to an accuracy of 64%; in the novel voice testing, they were 1.13 times as likely, corresponding to an accuracy of 53%. Greater performance in the familiar voice testing supports the HVPT findings that distinguishing words is easier when they are presented by familiar speakers [15, 16, 18]. Scores in the second test blocks were also on average lower than those in the first, suggesting either an effect of participant fatigue or interference from the first block. There is also evidence of the participants being able to generalize their training input to a novel speaker. Considering only the novel voice testing data presented in the first block, a linear mixed model with logit regression and no fixed effects and *participant identity* as a random effect had an intercept significantly greater than zero (*β* = 0.184, SE = 0.090, p = 0.041): participants were 1.20 times as likely to produce a correct response as incorrect, corresponding to an accuracy of 55%.

## Conclusions of Experiment 1

The lack of a difference between the conditions extends Saffran et al.’s [37] result to the case where the training data is presented by multiple voices, suggesting that segmentation of continuous speech may not be affected by the number of speakers who provide it. We have no evidence, however, that the effects of speaker input variability on phonemic acquisition can be extended to a learner’s ability to segment their linguistic input. Though the acquisition of morphology involves much more than segmenting input, determining word boundaries is still a crucial part of this process [57]. Therefore there is no evidence to support the proposal that speaker input variability could influence morphology learning.

## Experiment 2: learning morphology

Our first experiment, in assessing the effect of speaker input variability on the ability of a learner to isolate and identify individual morphemes in a speech stream, investigated a crucial part of an individual’s acquisition of a morphological system [37, 40]. But the learner has to do more than distinguish morpheme boundaries: they also have to relate the isolated components to meanings, be able to recombine them to create grammatically permissible utterances which convey particular semantic information, and then be able to produce these utterances. We conducted a second experiment which more closely reflects the full range of processes involved in morphology learning and so more thoroughly tests the effect of speaker input variability on the acquisition of morphology, assessing learner abilities to orally acquire a morphologically-complex miniature language.

## Materials and methods

This experiment was approved by the Linguistics and English Language Ethics Committee of the University of Edinburgh. Written consent was provided by all participants before taking part.

We asked participants to learn a miniature language based on 12 sentences of Hungarian. Hungarian has an extensive nominal case system in which nouns are (barring rare exceptions) obligatorily marked with case-indicating suffixes [58–60]. The particular form of a suffix is also often dependent on vowel harmony, with a [+back] feature in the initial vowel of the noun stem spreading throughout the stem and its suffixes [60–62]. Hungarian has 14 vowels, including a phonemic contrast between long and short vowels. The 6 [+back] vowels (with corresponding International Phonetic Alphabet representation) for the purposes of vowel harmony, are *a* (/ɔ/), *á* (/a:/), *o* (/o/), *ó* (/o:/), *u* (/u/) and *ú* (/u:/) [59]. For example, the inessive form of *város* /va:roʃ/, “city”, is *városban* /va:roʃbɔn/, “in the city”, while the corresponding form of *szék* /se:k/, “chair”, is *székben* /se:kbεn/, “in the chair” [58]. In the first case, the [+back] feature of *á* /a:/ spreads through the suffix, which takes the back vowel of *a* /ɔ/ in -*ban*, while in the second, the [-back] feature of *é* /e:/ results in the alternation -*ben* with the front vowel /ε/.

**Fig 3:**
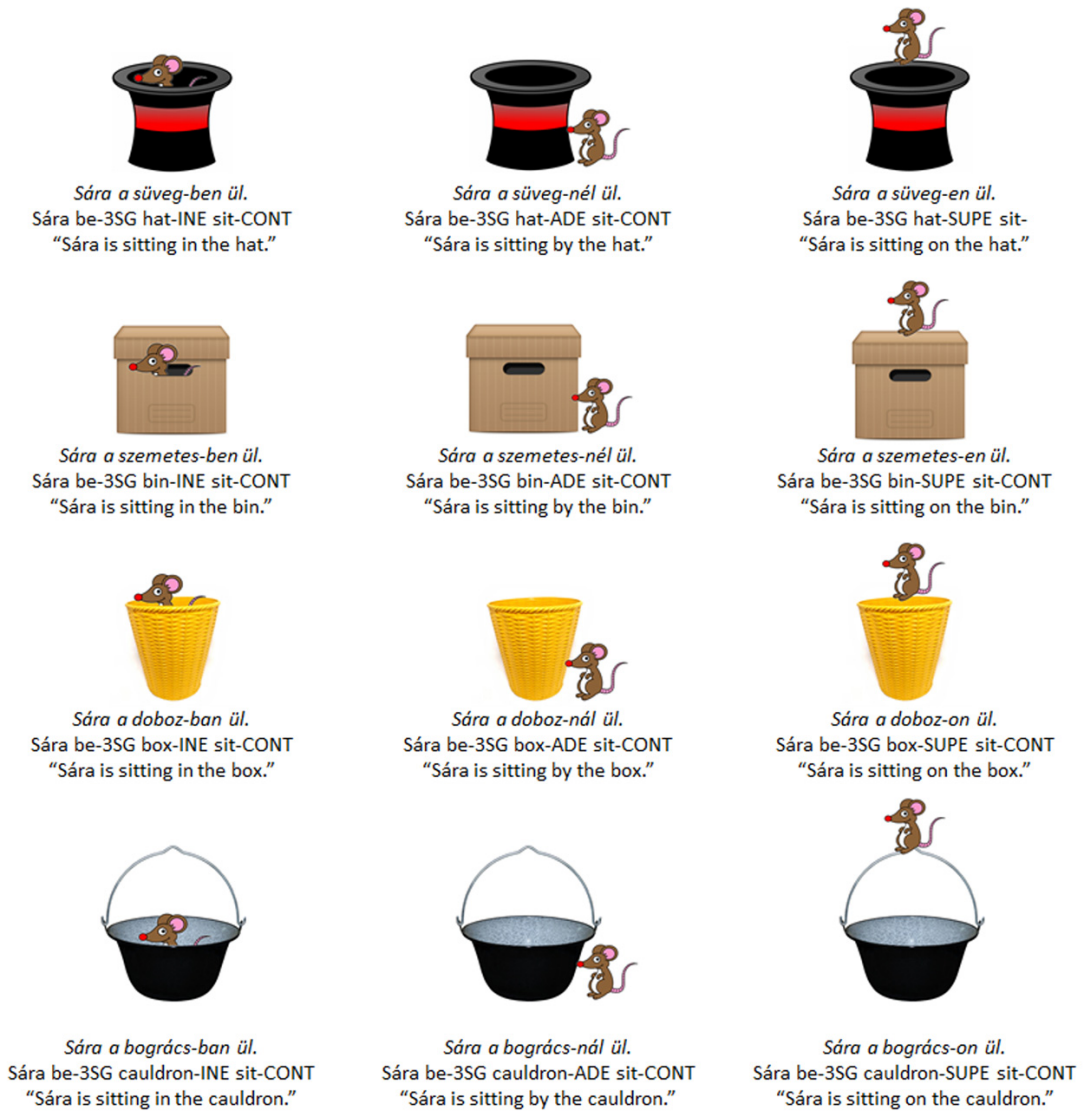
Complete target language with corresponding images.

Our target language used three cases: the inessive (“in”), adessive (“by” or “at”) and superessive (“on”). These were selected as they each require different affix variants dependent on the initial vowel in the noun stem [58] and were semantically easy to represent using simple and static visual stimuli. 12 images were created in which a cartoon mouse was shown located either in, next to, or on top of one of four containers: a hat, a wastepaper bin, a box and a cauldron. Two of the containers, *süveg* /ʃyvεg/ (“hat”) and *szemetes* /sεmεtεʃ/ (“bin”), have [-back] initial vowels, while the other two, *doboz* /doboz/ (“box”) and *bogrács* /bogra:tʃ/ (“cauldron”), have [+back]. The target language therefore includes semantically-redundant alternations within the case-marking affixes. Hungarian sentences describing each of the images then comprised the target language. The complete set of images and labels is given in Fig 3.

Three native speakers of Hungarian (1 female) were recruited to construct the aural training data. In an attempt to have as natural-sounding a stimuli set as possible, they were recorded producing each sentence three times, with the second production used in the experiment.

### Participants

40 participants (16 male; aged between 18 and 42, mean 21.4) were recruited using the Student and Graduate Employment (SAGE) database of the Careers Service of the University of Edinburgh, with non-native speakers of English and current and former students of linguistics excluded. Participants were asked to list the languages they could speak or understand, indicating their proficiency in each case. No applicants reported any prior knowledge of Hungarian or any other Uralic language. Participants were required to attend 3 sessions of approximately 20 minutes on consecutive days and at the same time each day. Each was compensated £12 on completion. Data for one further participant was rejected as they did not attend after the first session, and another participant was recruited in their place.

### Procedure

Each participant took part in 6 rounds of training and testing, 2 on each day. For each participant, 8 of the 12 target language sentences formed the training data, which were randomly selected with the constraints that two sentences described each container, that each case was represented at least twice and each alternation was represented at least once. The training data was therefore sufficient (in principle) to reconstruct the entire target language, including the 4 unseen sentences.

20 participants were randomly assigned to the single-speaker condition, where the 8 training sentences were produced by the same, randomly-selected speaker throughout the experiment. Each of the 3 speakers was assigned to at least 6 participants. In the multiple-speaker condition, the 8 training sentences were randomly assigned to the 3 speakers with the constraint that at least 2 sentences were presented by each speaker. Each training sentence was then presented by the same speaker throughout the experiment.

In each training round, the learner was exposed to 5 independently randomly sorted passes of the entire training set of 8 image-label pairings. For each item, the participant was first shown the image for 2 seconds in silence, before being played the appropriate audio file and then given 6 seconds to attempt to repeat what they had heard. Advance to the next item was automatic. Before the initial training stage, the learner was given two additional randomly-selected training items to check their comprehension of the task.

Each training stage was followed immediately by a test. The learner was required to orally label the entire set of 12 images (both the 8 seen in training and the 4 novel), presented in a random order. Once an image had been displayed for at least 3 seconds and the participant had had the opportunity to produce a label, any key press on the keyboard advanced the test to the next item.

The experiment was written and run in Matlab (R2010a) with the Psychtoolbox extensions. Audio data was collected using the ProTools LE software and the Digidesign 003 audio interface.

## Analysis and results

### Production of the noun stems

For each participant utterance, the noun stem and case-marking suffix were segmented and transcribed using the following phoneme set: /y, ε, a, ɔ, ə, m, n, ŋ, b, p, d, t, g, k, f, v, s, ʃ, z, ʒ, $\overset{⌢}{\text{t}ʃ}$, $\overset{⌢}{\text{d}ʒ}$, w, l, r, j/. Due to hesitations and pauses in the productions, it was not possible to transcribe meaningful length distinctions. Production of the noun stems was then assessed by considering a modified normalised weighted Levenshtein edit distance between the produced stem and target, with distance from individual phonemes based on the articulatory feature values provided by Connolly [63]. Feature values for the vowels and consonants of our transcription set are given in Tables 1 and 2, respectively. We have assumed that all unvoiced plosives are aspirated, have set the sulcral values for /ʒ/, /$\overset{⌢}{\text{t}ʃ}$/, /$\overset{⌢}{\text{d}ʒ}$/, /w/, /l/, /r/ and /j/ ourselves, and have taken average values for double articulators.

**Table 1:** Articulatory feature values for vowels.

Vowels	/y/	/ε/	/a/	/ɔ/	/u/	/ə/
Height	1	0.5	0	0.5	1	0.5
Forwardness	1	1	0.5	0	0	0.5

**Table 2:** Articulatory feature values for consonants.

Consonants	n	m	ŋ	b	p	d	t	g	k	f	v	s	ʃ	z	ʒ	$\overset{⌢}{\text{t}ʃ}$	$\overset{⌢}{\text{d}ʒ}$	w	l	r	j
Aspiration	0	0	0	0	1	0	1	0	1	0.5	0	0.5	0.5	0	0	0.5	0	0	0	0	0
Place	0.85	1	0.6	1	1	0.85	0.85	0.6	0.6	0.9	0.9	0.85	0.8	0.85	0.8	0.85	0.85	0.8	0.85	0.8	0.7
Constrictor	0.85	1	0.6	1	1	0.85	0.85	0.6	0.6	1	1	0.85	0.85	0.85	0.85	0.85	0.85	0.8	0.85	0.85	0.6
Stop	1	1	1	1	1	1	1	1	1	0.9	0.9	0.9	0.9	0.9	0.9	0.95	0.95	0	0	0	0
Nasal	1	1	1	0	0	0	0	0	0	0	0	0	0	0	0	0	0	0	0	0	0
Lateral	0	0	0	0	0	0	0	0	0	0	0	0	0	0	0	0	0	0	1	0	0
Sulcal	0	0	0	0	0	0	0	0	0	0	0	1	0.8	1	0.8	0.8	0.8	0	0	0	0
Double	0	0	0	0	0	0	0	0	0	0	0	0	0	0	0	1	1	0	0	0	0

Following the recommendations of previous work [64, 65], insertions and deletions were given an edit cost of 1, and replacement of a vowel with a vowel or a consonant with a consonant a maximum value of 0.8. Replacing a vowel with a consonant or vice versa incurred a cost of 1. The distance between two phonemes was calculated by taking the sum of the absolute values between each of their features. So, for example, the distance between /y/ and /a/ is calculated by |1 − 0| + |1 − 0.5| = 1.5, and the distance between /n/ and /$\overset{⌢}{\text{t}ʃ}$/ by |0 − 0.5| + |0.85 − 0.85| + |0.85 − 0.85| + |1 − 0.95| + |1 − 0| + |0 − 0| + |0 − 0.8| + |0 − 1| = 3.35. These distances are then normalised by dividing by the maximum distance within the set of vowels (1.5) or consonants (4.25), and then multiplying by the maximum within-category phoneme replacement factor of 0.8 [64]. A final distance between two strings was then normalised by the length of the longer string, and an accuracy score calculated as 1 minus this value.

For example, consider the distance between the two strings /kam/ and /fi/. Replacing /k/ with /f/ incurs a cost of (1.3/4.25) × 0.8. Replacing /a/ with /i/ incurs a cost of (1.5/1.5) × 0.8 (note that this is the maximum distance between two vowels). Inserting /m/ incurs a cost of 1. Normalising the sum by dividing by the maximum string length of 3, we have a distance measure of 0.682, and so an accuracy score of 1–0.682 = 0.328.

**Fig 4:**
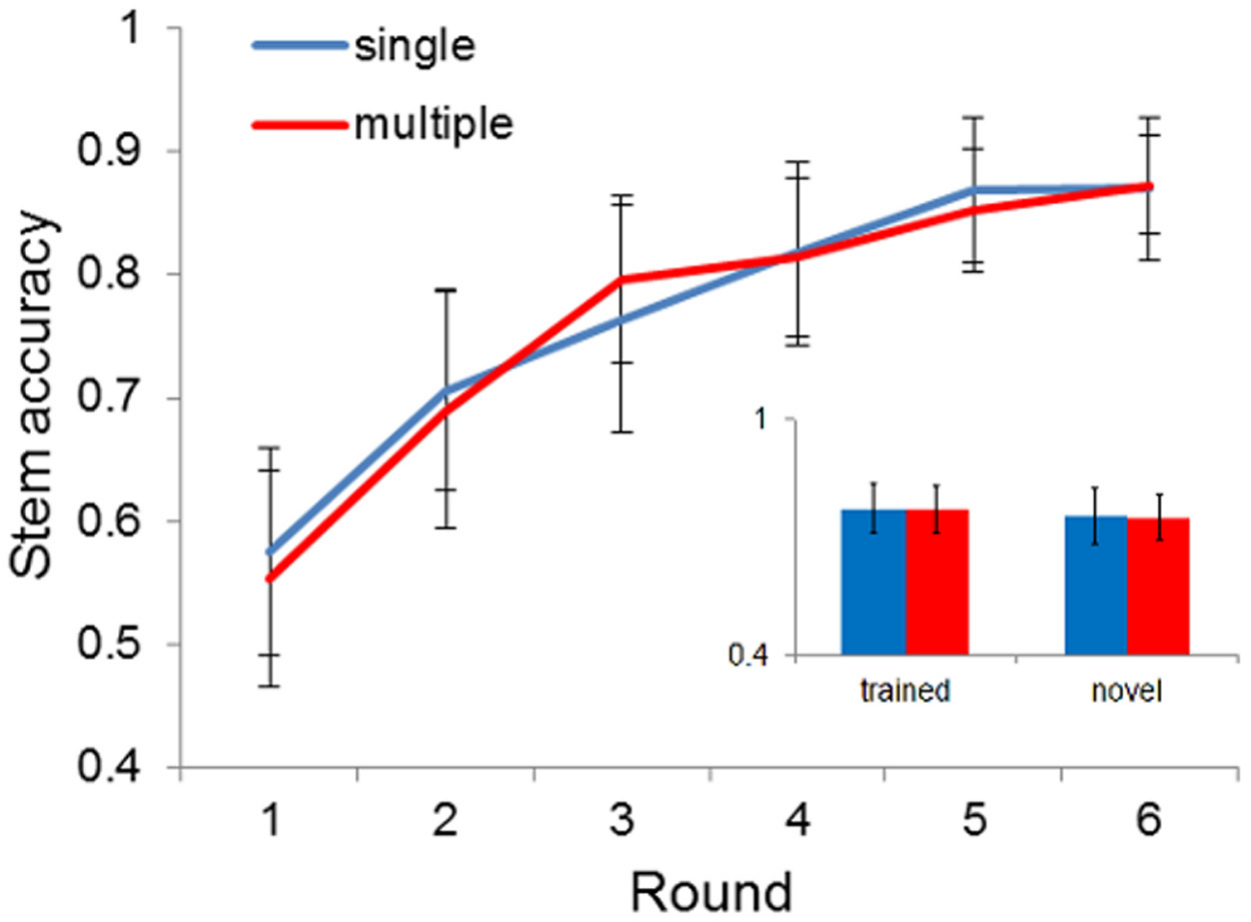
Accuracy of participant productions of target stems. Main graph shows the production scores of the complete target language (the entire set of 12 items). The insert illustrates the minimal difference between the average scores (over all rounds) for the trained and novel items. Error bars represent 95% confidence intervals of the mean.

Mean stem accuracy for each of the conditions over the 6 rounds is illustrated in Fig 4.

We performed a linear mixed effects analysis using R [54] and *lme4* [55]. A maximal model [56] included *condition* (single speaker or multiple speaker), *novelty* (whether the target stimulus had been seen in training or not) and *round* and their interactions as (centred) fixed effects. *Participant identity* was investigated as a random effect. This model was significantly better than the equivalent null model (*χ* ^2^(7) = 628.91, p <0.001). P-values were estimated from the resultant t-statistics with 2873 degrees of freedom, the number of observations minus the number of fixed parameters in the model [66]. There were significant effects of *round* (*β* = 0.059, SE = 0.002, t (2873) = 26.35, p <0.001) and *novelty* (*β* = 0.020, SE = 0.008, t (2873) = 2.40, p = 0.016), but no effect of *condition* (*β* = 0.004, SE = 0.041, t (2873) = 0.09, p = 0.928) or any of the interaction terms.

This analysis suggests that participant production of the noun stems improved with increased training and testing, and that participants more accurately produced the stems for images they saw in training. No effect of condition suggests that speaker input variability had no effect on acquisition. There is therefore no evidence that the number of speakers who provide the input affects language acquisition in general, and we turn our attention to assessing the claim that it may have a specific effect on morphology.

### Production of the affixes

To assess participant acquisition of the morphological system, each produced affix was binary coded using three increasingly stringent measures:

1. Case identification—“1” if and only if the affix unambiguously identified the correct case of the target.
2. Case accuracy—“1” if and only if the affix was an accurate reproduction of one of the alternations for the case of the target.
3. Alternation accuracy—“1” if and only if the affix was an accurate reproduction of the correct, vowel-harmony dependent, alternation of the target.

For example, consider the target suffix for a [-back] stem marking the inessive case, -*ben* /-bεn/. A production of /-bεm/ would be coded 1 for case identification, 0 for case accuracy and 0 for alternation accuracy, as while the target case can be unambiguously recovered from the production, the realisation does not exactly match either suffix (corresponding to either [-back] or [+back] stems) which marks the inessive case in the target language. A production of /-bɔn/ would be coded 1 for case identification and 1 for case accuracy, as although the alternation is not appropriate for a [-back] stem, the participant accurately produced one of the suffixes of the target cases, but violated vowel harmony. Only a production of /-bεn/ would score 1 for all three measures.

The coding for each of the measures was carried out twice. The measurements were first hand-coded directly from the recordings of the participants’ productions. These were then compared to calculations of modified normalised weighted Levenshtein edit distances between the transcriptions of the produced affixes and the affixes of the target language calculated using the same methods as described for the stems above. For the case identification measure, we calculated the edit distances between the transcription and each of the 6 suffixes of the whole target language. We then checked that a score of 1 had been coded if and only if the lowest of these edit distance corresponded to the distance between the transcription and one of the two suffixes of the target case. For example, if the target was in the inessive case, we confirmed that a score of 1 was awarded if and only if the edit distance between the transcription and /-bεn/ or the edit distance between the transcription and /-bɔn/ was lower than all the other distances between the transcription and the other suffixes of the language. For the case accuracy measure, we checked that a hand-coded score of 1 corresponded to the edit distance between the transcription and one of the two suffixes of the target case being 0. For the alternation accuracy measure, we checked that a hand-coded score of 1 corresponded to the edit distance between the transcription and the target suffix being 0.

**Fig 5:**
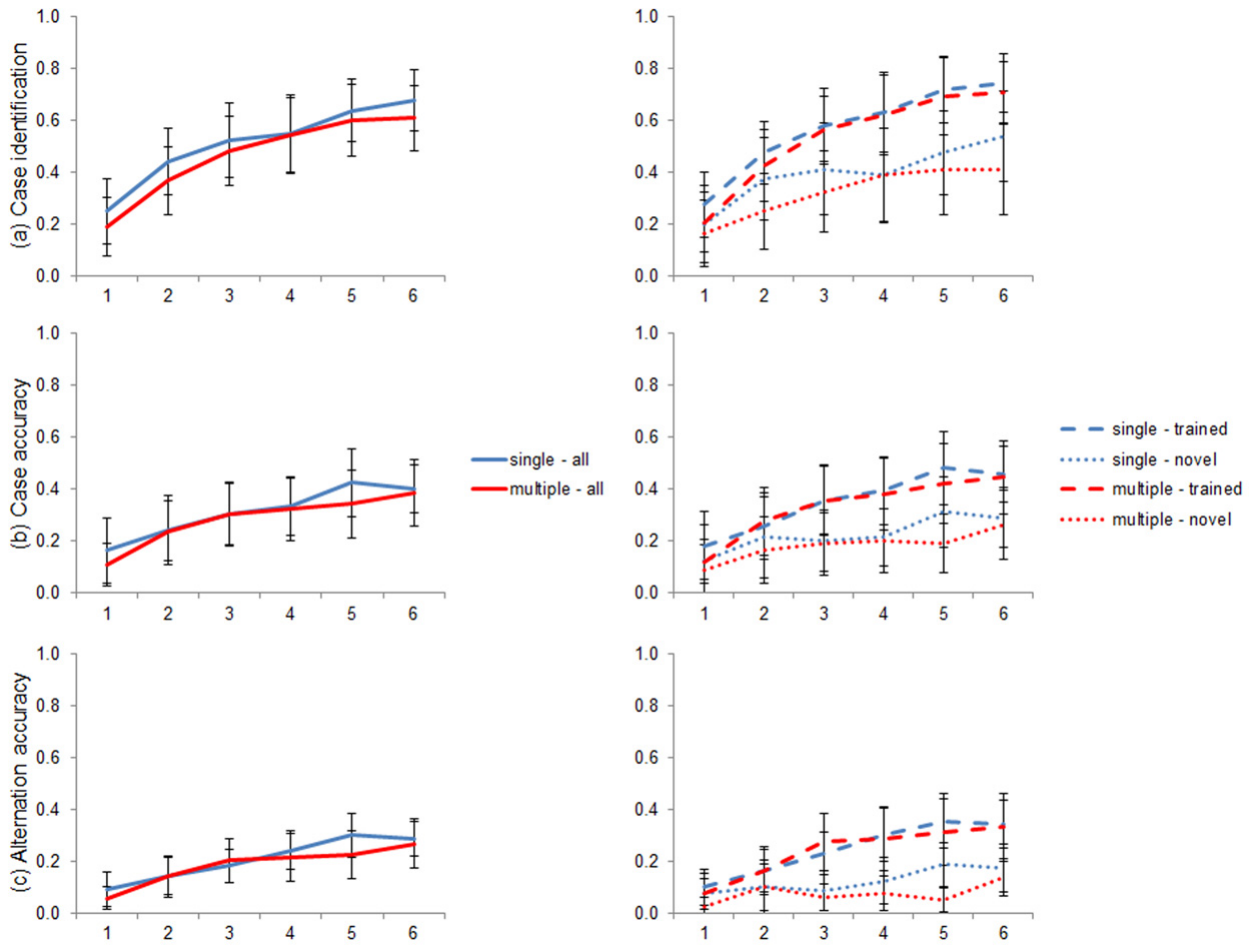
Acquisition of the suffixes. Mean scores by condition are shown for each of the 3 measures, both for the entire target language set (left), and split by training and novel image labels. Error bars show 95% confidence intervals of the mean.

The results by condition for each measure are shown in Fig 5. Average scores for the whole language are given, along with a comparison of the scores relating to the trained and the novel images.

We performed linear mixed analyses for each measure, using logit regression and maximal models [56] which again included *condition*, *novelty* and *round* and their interactions as (centred) fixed effects. *Participant identity* was again included as a random effect. For all three measures, the fitted model was better than the corresponding null model (Case identification: *χ* ^2^(7) = 434.12, p <0.001; Case accuracy: *χ* ^2^(7) = 218.17, p <0.001; Alternation accuracy: *χ* ^2^(7) = 216.71, p <0.001).

For the case identification measure, there were significant effects of *novelty* (*β* = 1.112, SE = 0.100, p <0.001), *round* (*β* = 0.464, SE = 0.029, p <0.001) and the interaction of *novelty* and *round* (*β* = 0.203, SE = 0.059, p <0.001). There was no effect of *condition* (*β* = 0.390, SE = 0.482, p = 0.419) or any of the other interaction terms (p ≥ 0.166):

For the case accuracy measure, there were significant effects of *novelty* (*β* = 0.936, SE = 0.110, p <0.001) and *round* (*β* = 0.321, SE = 0.029, p <0.001), and an approaching significance effect of the interaction of *novelty* and *round* (*β* = 0.123, SE = 0.064, p = 0.055). There was no significant effect of *condition* (*β* = 0.354, SE = 0.465, p = 0.447), or any of the other interaction terms (p ≥ 0.327).

For the alternation accuracy measure, there were significant effects of *novelty* (*β* = 1.223, SE = 0.134, p <0.001) and *round* (*β* = 0.300, SE = 0.033, p <0.001). There was no significant effect of *condition* (*β* = 0.468, SE = 0.389, p = 0.226) or any of the interaction terms (p ≥ 0.276).

## Conclusions of Experiment 2

Whichever measure we consider, this analysis indicates that participant affix productions improved with increased training and testing, and that the labelling of novel images was worse than that of those seen in training. As in Experiment 1, we find no evidence to support a hypothesis that speaker input variability aids language acquisition, and so again have no support for the suggestion that it should be considered a mechanism by which group size can determine a language’s morphological complexity.

## Discussion

These experiments provide no evidence to support the hypothesis that speaker input variability may influence language learning beyond the acquisition of phonemic [15, 16, 18, 19] or tonal [13] distinctions. We cannot, of course, rule out the possibility that such variability does affect the acquisition of a morphological system, but that we have failed to capture it. The contrast between our conditions may have been too slight, our samples sizes too small, or our assessment measures too crude. Our experiments may also lack sufficient ecological validity. For obvious reasons of practicality and control, we have attempted to investigate natural language-learning process using adult participants in an artificial laboratory setting. This constitutes an important caveat on our interpretation of our results, particularly in light of some evidence that children may respond to input variability differently to adults [67].

To address such concerns, these experiments could be adapted and extended in a number of ways. The contrast between conditions could be increased simply by having a greater number of speakers in the multiple speaker conditions (for comparison, higher variability in HVPT studies is typically represented by 5 speakers [15, 16, 18, 19]), or the homogeneity of the input could also have been decreased in the multiple-speaker conditions in other ways. Speech-rate differences could have been included in Experiment 1, for example, or a language with a greater amount of inter-speaker variation in pronunciation could have been used to construct the target language in Experiment 2 (Hungarian being notably uniform across its dialects [58]). If the proposed effect is relatively subtle, our experiments may also be improved by larger sample sizes, increased training and testing, or by studying the acquisition of a much larger target language ([46] illustrates how Experiment 1 could be made more challenging by increasing the number and length of target items and reducing training). Frank et al.’s study [40], for example, could be adapted to include a multiple speaker condition. As demonstrated by Saffran et al. [38], adapting Experiment 1 in particular to study the effects in infants children would also be a possibility.

While we would welcome future experimental work in this area, the results of these two experiments do suggest that the speaker input variability effect of phoneme and toneme acquisition cannot (transparently at least) be extended beyond the findings of the HVPT studies, and that it is therefore unlikely to be an explanatory mechanism for how group size determines a language’s morphological complexity. We have the same null result in two different experiments, which consider two different stages of the language acquisition process, involve both artificial and natural language learning, and test word segmentation in reception and morphological generalisation in production. Our replication of previous results [37] in the familiar voice test of Experiment 1 in both conditions also suggests that our experimental design and procedure were appropriate, that the participants interpreted the task as intended, and therefore that the result of the second condition is valid. There is also no indication that participants misunderstood the task or adopted particularly obscure strategies in Experiment 2. In a post-experiment interview, 39 of the 40 participants reported their attempts to parse the training sentences to determine which segment corresponded to the container and which to the position of the mouse in the images (the remaining participant said that they would have followed this approach if they had believed that they would have been able to do so successfully in the time available). No participant reported not being able to detect a difference between the training sentences.

If speaker input variability does not affect an individual’s learning of morphology, then where does this leave the proposal that input variability could explain how group size determines a language’s morphological complexity? One possibility is that increased speaker input variability only limits the cross-generational transfer of morphology when “morphological distinctions rely on a single segment or even sub-segmental phonological change”, which is often the case in natural languages [5, p. 1833]. Acquisition difficulties would then arise from learners not being able to detect a difference between minimally different input strings (which was not an issue for the learners in our second experiment). This would suggest, however, that speaker input variability could only be a partial explanation of why languages spoken by more people are simpler. Another possibility is that some type of input variability does have an effect on cross-generational transfer of morphology, but not that which arises at the level of phoneme realisation. Syntactic or lexical variability, for example, may be higher in larger groups and result in simplification across generations of transmission. The predictability of such variability and how it is distributed across speakers would then probably be important factors in determining its effects [68, 69], as would the age of learners who receive it [67]. This is certainly worth further investigation.

It is also worth commentating that even if any effects of input variability (in any form) on language learning can be demonstrated, accounting for how such individual effects can result in language-level change is not necessarily trivial [6], while a convincing demonstration of how and why input variability in larger groups is actually greater is also necessary. We accept that the presumption that an individual’s social network is likely to be larger in a larger group is reasonable. However, this may not impact on the variability of the input *which is relevant to language acquisition*, given the influence of other sociocultural factors, such as family size and the role of each parent in childcare [70].

Given these issues and the null results of the experiments, it is worth considering other explanations for how group size could influence morphological complexity. Two other candidate mechanisms are discussed by Nettle [5].

One possibility is that (cultural) drift, which has a more pronounced effect in smaller populations [71], may cause faster rates of linguistic change which result in groups adopting “suboptimal” communicative strategies, such as more complex, overspecified, morphological systems [36, 72]. There are a number of problems with such an explanation, however, not least empirical evidence suggesting that linguistic change may actually be slower in smaller populations [5].

An alternative considers the effect non-native learners can have on a language. Languages spoken by a greater number of people appear to have a greater number of non-native speakers [10]. Older learners are also thought to find the acquisition of complex morphology more challenging compared to other means of encoding the same semantic information. More widely spoken languages might therefore be under similar pressures as those in language contact situations [36]. They will simplify grammatically as they adapt to the needs and preferences of their non-native speakers: “difficult” language features will be filtered out, and more transparent, lexical strategies will be favoured over morphological ones [2, 10, 36, 73, 74]. This in turn leads to a greater reliance on extralinguistic, pragmatic, information, which is again better suited to adult learners [10, 75–77].

A challenge for this account, however, is the focus on simplification of languages due to adult learning: arguably it must also account for the relative complexity of languages with fewer non-native learners [5]. One proposal is that the complex(ified) nature of smaller languages reflects some “default” psycholinguistic state of its speakers, which will be reverted to in the absence of pressures resulting from more exoteric communication [2]. Alternatively, if pressures for language simplification are relaxed, more complex, morphological, strategies may be favoured over syntactic ones in the interests of conciseness and efficiency [3, 5]. Another suggestion is that added complexity in the form of grammatical redundancy may actually aid child language acquisition [10]. It may compensate for the difficulties children have in using pragmatic inference to resolve ambiguous utterances [75–77], or by providing more evidence as to how the signal should be segmented [5, 10]. Further work would be necessary to support such claims [5].

## Conclusion

The two experiments described here offer no support for the proposal that speaker input variability can affect the acquisition of morphology. In our first experiment, assessing the ability of adult learners to segment continuous input streams using only the transitional probabilities between syllables, participants were able to discriminate between the words of the training data and foils regardless of whether the input was provided by a single speaker or three. This extends previous work assessing the ability of learners to use distribution cues to parse input data [37] to a case where the input is provided by multiple speakers.

The second experiment, which assessed the acquisition of a miniature language with case-marking affixes, also found no affect of speaker input variability. Therefore we have no evidence to support the proposal that such variability may be a causal explanation for the link between group size and morphological complexity [5, 10]. Given these experimental results, and doubts about the proposed relationship between population size and input variability, we ultimately suggest that it is probably not. We would of course still welcome further tests of speaker input variability’s effects, although do believe that investigation of alternative explanations for proposed sociocultural determination of linguistic complexity would be more fruitful.

## References

[1] Christiansen MH, Chater N. Language as shaped by the brain. Behav Brain Sci. 2008;31: 489–508; discussion 509–558. 18826669 10.1017/S0140525X08004998

[2] Wray A, Grace GW. The consequences of talking to strangers: Evolutionary corollaries of socio-cultural influences on linguistic form. Lingua. 2007;117: 543–578. 10.1016/j.lingua.2005.05.005

[3] Trudgill P. Sociolinguistic Typology: Social Determinants of Linguistic Complexity. Oxford: Oxford University Press; 2011.

[4] Dale R, Lupyan G. Understanding the Origins of Morphological Diversity: the Linguistic Niche Hypothesis. Adv Complex Syst. 2012;15: 1150017 10.1142/S0219525911500172

[5] Nettle D. Social scale and structural complexity in human languages. Philos Trans R Soc Lond B Biol Sci. 2012;367: 1829–1836. 10.1098/rstb.2011.0216 22641821 PMC3367698

[6] Kirby S. Function Selection and Innateness: the Emergence of Language Universals. Oxford: Oxford University Press; 1999.

[7] Smith K, Kirby S. Cultural evolution: implications for understanding the human language faculty and its evolution. Philos Trans R Soc Lond B Biol Sci. 2008;363: 3591–3603. 10.1098/rstb.2008.0145 18801718 PMC2607345

[8] Smith K. Evolutionary perspectives on statistical learning In: Rebuschat P, Williams JN, editors. Statistical Learning and Language Acquisition. Berlin: De Gruyter Mouton; 2012 pp. 409–432.

[9] Evans N, Levinson SC. The myth of language universals: language diversity and its importance for cognitive science. Behav Brain Sci. 2009;32: 429–48; discussion 448–492. 10.1017/S0140525X0999094X 19857320

[10] Lupyan G, Dale R. Language structure is partly determined by social structure. PLoS One. 2010;5: e8559 10.1371/journal.pone.0008559 20098492 PMC2798932

[11] Mullennix JW, Pisoni DB, Martin CS. Some effects of talker variability on spoken word recognition. J Acoust Soc Am. 1989;85: 365–378. 10.1121/1.397688 2921419 PMC3515846

[12] Logan JS, Lively SE, Pisoni DB. Training Japanese listeners to identify English /r/ and /l/: a first report. J Acoust Soc Am. 1991;89: 874–886. 10.1121/1.1894649 2016438 PMC3518834

[13] Wang Y, Spence MM, Jongman A, Sereno JA. Training American listeners to perceive Mandarin tones. J Acoust Soc Am. 1999;106: 3649–3658. 10.1121/1.428217 10615703

[14] Iverson P, Hazan V, Bannister K. Phonetic training with acoustic cue manipulations: A comparison of methods for teaching English /r/-/l/ to Japanese adults. J Acoust Soc Am. 2005;118: 3267–3278. 10.1121/1.2062307 16334698

[15] Lively SE, Pisoni DB, Yamada RA, Tohkura Y, Yamada T. Training Japanese listeners to identify English /r/ and /l/. III. Long-term retention of new phonetic categories. J Acoust Soc Am. 1994;96: 2076–2087. 10.1121/1.410149 7963022 PMC3518835

[16] Bradlow AR, Akahane-Yamada R, Pisoni DB, Tohkura Y. Training Japanese listeners to identify English /r/ and /l/: Long-term retention of learning in perception and production. J Acoust Soc Am. 1999;61: 977–985. 10.3758/bf03206911 PMC3472521 10499009

[17] Hirata Y, Whitehurst E, Cullings E. Training native English speakers to identify Japanese vowel length contrast with sentences at varied speaking rates. J Acoust Soc Am. 2007; 121: 3837–3845. 10.1121/1.2734401 17552731

[18] Lively SE, Logan JS, Pisoni DB. Training Japanese listeners to identify English /r/ and /l/. II: The role of phonetic environment and talker variability in learning new perceptual categories. J Acoust Soc Am. 1993;94: 1242–1255. 10.1121/1.408177 8408964 PMC3509365

[19] Bradlow AR, Pisoni DB, Akahane-Yamada R, Tohkura Y. Training Japanese listeners to identify English /r/ and /l/: IV. Some effects of perceptual learning on speech production. J Acoust Soc Am. 1997;101: 2299–2310. 10.1121/1.418276 9104031 PMC3507383

[20] Petersen AM, Tenenbaum JN, Havlin S, Stanley HE, Perc M. Languages cool as they expand: Allometric scaling and the decreasing need for new words. Sci Rep. 2012;2 943 10.1038/srep00943 23230508 PMC3517984

[21] Fought JG, Munroe RL, Fought CR, Good EM. Sonority and climate in a world sample of languages. Cross-Cultural Res. 2004;38: 27–51. 10.1177/1069397103259439

[22] Ember CR, Ember M. Climate, econiche, and sexuality: Influences on sonority in language. Am Anthropol. 2007;109: 180–185. 10.1525/aa.2007.109.1.180

[23] Munroe RL, Fought JG, Macaulay RKS. Warm climates and sonority classes: Not simply more vowels and fewer consonants. Cross-Cultural Res. 2009;43: 123–133. 10.1177/1069397109331485

[24] Atkinson QD. Phonemic diversity supports a serial founder effect model of language expansion from Africa. Science. 2011;332: 346–349. 10.1126/science.1199295 21493858

[25] Hay J, Bauer L. Phoneme inventory size and population size. Language. 2007;83: 388–400. 10.1353/lan.2007.0071

[26] Wichmann S, Rama T, Holman EW. Phonological diversity, word length, and population sizes across languages: The ASJP evidence. Linguist Typology. 2011;15: 177–197.

[27] Jackendoff R. Possible stages in the evolution of the language capacity. Trends Cogn Sci. 1999;3: 272–279. 10.1016/S1364-6613(99)01333-9 10377542

[28] Nichols J. Linguistic complexity: a comprehensive definition and survey In: Sampson G, Gil D, Trudgill P, editors. Language Complexity as an Evolving Variable. Oxford: Oxford University Press; 2009 pp. 110–125.

[29] Sinnemäki K. Complexity in core argument marking and population size In: Sampson G, Gil D, Trudgill P, editors. Language Complexity as an Evolving Variable. Oxford: Oxford University Press; 2009 pp. 126–140.

[30] Haspelmath M, Dryer M, Gil D, Comrie B. The World Atlas of Language Structures Online. n.d. Munich: Max Plank Digital Library; Available: http://wals.info/

[31] Reali F, Chater N, Christiansen MH. The paradox of linguistic complexity and community size. In: Cartmill, EA, Roberts, S, Lyn, H, Cornish, H, editors. Proceedings of the 10th International Conference on the Evolution of Language (EVOLANG X). Singapore: World Scientific Publishing Co. Pte. Ltd.; 2014. pp. 270–279.

[32] Perkins RD. Deixis, Grammar and Culture. Amsterdam: Benjamins; 1992.

[33] Martowicz A. The origin and functioning of circumstantial clause linkers: a cross-linguistic study. Ph.D. Thesis, University of Edinburgh. 2011.

[34] Maas U. Orality versus literacy as a dimension of complexity In: Sampson G, Gil D, Trudgill P, editors. Language Complexity as an Evolving Variable. Oxford: Oxford University Press; 2009 pp. 164–177.

[35] McWhorter JH. Defining creole. Oxford: Oxford University Press; 2005.

[36] Trudgill P. Linguistic and social typology: The Austronesian migrations and phoneme inventories. Linguist Typology. 2004;8: 305–320. 10.1515/lity.2004.8.3.305

[37] Saffran JR, Newport EL, Aslin RN. Word segmentation: the role of distributional cues. J Mem Lang. 1996;621: 606–621. 10.1006/jmla.1996.0032

[38] Saffran JR, Aslin RN, Newport EL. Statistical learning by 8-month-old infants. Science. 1996;274: 1926–1928. 10.1126/science.274.5294.1926 8943209

[39] Johnson EK, Jusczyk PW. Word Segmentation by 8-Month-Olds: When Speech Cues Count More Than Statistics. J Mem Lang. 2001;44: 548–567. 10.1006/jmla.2000.2755

[40] Frank MC, Tenenbaum JB, Gibson E. Learning and long-term retention of large-scale artificial languages. PLoS One. 2013;8: e52500 10.1371/journal.pone.0052500 23300975 PMC3534673

[41] Weiss DJ, Gerfen C, Mitchel AD. Speech segmentation in a simulated bilingual environment: a challenge for statistical learning? Lang Learn Dev. 2014;5: 30–49. 10.1080/15475440802340101 PMC3981102 24729760

[42] Saffran JR, Johnson EK, Aslin RN, Newport EL. Statistical learning of tone sequences by human infants and adults. Cognition. 1999;70: 27–52. 10.1016/S0010-0277(98)00075-4 10193055

[43] Fiser J, Aslin RN. Unsupervised Statistical Learning of Higher-Order Spatial Structures from Visual Scenes. Psychol Sci. 2001;12: 499–504. 10.1111/1467-9280.00392 11760138

[44] Kirkham NZ, Slemmer JA, Johnson SP. Visual statistical learning in infancy: evidence for a domain general learning mechanism. Cognition. 2002;83: B35–B42. 10.1016/S0010-0277(02)00004-5 11869728

[45] Hauser MD, Newport EL, Aslin RN. Segmentation of the speech stream in a non-human primate: statistical learning in cotton-top tamarins. Cognition. 2001;78: B53–B64. 10.1016/S0010-0277(00)00132-3 11124355

[46] Frank MC, Goldwater S, Griffiths TL, Tenenbaum JB. Modeling human performance in statistical word segmentation. Cognition.; 2010;117: 107–125. 10.1016/j.cognition.2010.07.005 20832060

[47] Dutoit T, Pagel V, Pierret N, Bataille F, van der Vrecken O. The MBROLA project: towards a set of high quality speech synthesizers free of use for non commercial purposes. Proceedings of the Fourth International Conference on Spoken Language. 1996. pp. 1393–1396.

[48] Thiessen ED, Saffran JR. When cues collide: Use of stress and statistical cues to word boundaries by 7- to 9-month-old infants. Dev Psychol. 2003;39: 706–716. 10.1037/0012-1649.39.4.706 12859124

[49] Yang J, Li P. Brain networks of explicit and implicit learning. PLoS ONE. 2012;7: e42993 10.1371/journal.pone.0042993 22952624 PMC3432050

[50] Witt A, Puspitawati I, Vinter A. How explicit and implicit test instructions in an implicit learning task affect performance. PLoS ONE. 2013;8: e53296 10.1371/journal.pone.0053296 23326409 PMC3541178

[51] Finn AS, Lee T, Kraus S, Hudson Kam CL. When it hurts (and helps) to try: the role of effort in language learning. PLoS ONE. 2014; 9: e101806 10.1371/journal.pone.0101806 25047901 PMC4105409

[52] Kachergis G, Yu C, Shiffrin RM. Cross-situational word learning is both implicit and strategic. Front Psychol. 2014;5: 588 10.3389/fpsyg.2014.00588 24982644 PMC4055842

[53] Arciuli J, Torkildsen J von K, Stevens DJ, Simpson IC. Statistical learning under incidental versus intentional conditions. Front Psychol. 2014;5: 747 10.3389/fpsyg.2014.00747 25071692 PMC4091029

[54] Core Team R. R: A language and environment for statistical computing. Vienna, Austria: R Foundation for Statistical Computing; 2013 Available: http://www.r-project.org/

[55] Bates D, Maechler M, Bolker B. lme4: Linear mixed-effects models using S4 classes. 2013. Available: http://cran.r-project.org/package=lme4

[56] Barr DJ, Levy R, Scheepers C, Tily HJ. Random effects structure for confirmatory hypothesis testing: Keep it maximal. J Mem Lang; 2013;68: 255–278. 10.1016/j.jml.2012.11.001 PMC3881361 24403724

[57] Finn A, Hudson Kam CL. Why segmentation matters: Experience-driven segmentation errors impair “morpheme” learning. J Exp Psychol Learn Mem Cogn; 2015 3 2 [Epub ahead of print] 10.1037/xlm0000114 25730305 PMC4558406

[58] Kenesei I, Vago RM, Fenyvesi A. Hungarian. London: Routledge; 1998.

[59] Siptár P, Törkenczy M. The Phonology of Hungarian. Oxford: Oxford University Press; 2007.

[60] Hayes B, Londe ZC. Stochastic phonological knowledge: the case of Hungarian vowel harmony. Phonology. 2006;23: 59–104. 10.1017/S0952675706000765

[61] Ringen CO, Vago RM, Ringen C. Hungarian vowel harmony in optimality theory. Phonology. 1998;15: 393–416. 10.1017/S0952675799003632

[62] Beňuš Š, Gafos A, Goldstein L. Phonetics and Phonology of Transparent Vowels in Hungarian. In: Nowak PM, Yoquelet C, Mortensen D, editors. Proceedings of the 3rd Speech Prosody Conference. 2004. pp. 486–497.

[63] Connolly JH. Quantifying target-realization differences. Part I: Segments. Clin Linguist Phon. 1997; 11: 267–287. 10.3109/02699209708985196

[64] Connolly JH. Quantifying target-realization differences. Part 11: Sequences. Clin Linguist Phon. 1997;11: 289–298. 10.3109/02699209708985196

[65] Sullivan J, McMahon A. Phonetic comparison, varieties, and networks: Swadesh’s influence lives on here too. Diachronica. 2010;27:325–340. 10.1075/dia.27.2.08sul

[66] Baayen RH, Davidson DJ, Bates DM. Mixed-effects modeling with crossed random effects for subjects and items. J Mem Lang;59: 390–412. 10.1016/j.jml.2007.12.005

[67] Hudson Kam CL, Newport EL. Getting it right by getting it wrong: when learners change languages. Cogn Psychol; 2009;59: 30–66. 10.1016/j.cogpsych.2009.01.001 19324332 PMC2703698

[68] Smith K, Wonnacott E. Eliminating unpredictable variation through iterated learning. Cognition; 2010;116: 444–449. 10.1016/j.cognition.2010.06.004 20615499

[69] Feher O, Kirby S, Smith K. Social influences on the regularization of unpredictable variation. Proceedings of the 36th Annual Conference of the Cognitive Science Society. Austin, TX: Cognitive Science Society; 2014. pp. 2187–2191.

[70] Barton ME, Tamasello M. The rest of the family: the role of fathers and siblings in early language development In: Galloway C, Richards BJ, editors. Input and Interaction in Language Acquisition. Cambridge: Cambridge University Press; 1994 pp. 109–134.

[71] Boyd R, Richerson PJ. Culture and the Evolutionary Process. Chicago: University of Chicago Press; 1985.

[72] Nettle D. Is the rate of linguistic change constant? Lingua. 1999;108: 119–136. 10.1016/S0024-3841(98)00047-3

[73] Dahl Ö. The Growth and Maintenance of Linguistic Complexity. Amsterdam: John Benjamins; 2004.

[74] Bentz C, Winter B. Languages with more second language learners tend to lose nominal case. Lang Dyn Chang. 2013;3: 1–27.

[75] Trueswell JC, Sekerina I, Hill NM, Logrip ML. The kindergarten-path effect: studying on-line sentence processing in young children. Cognition. 1999;73: 89–134. 10.1016/S0010-0277(99)00032-3 10580160

[76] Snedeker J, Trueswell JC. The developing constraints on parsing decisions: the role of lexical-biases and referential scenes in child and adult sentence processing. Cogn Psychol. 2004;49:238–299. 10.1016/j.cogpsych.2004.03.001 15342261

[77] Weighall AR. The kindergarten path effect revisited: children’s use of context in processing structural ambiguities. J Exp Child Psychol. 2008;99: 75–95. 10.1016/j.jecp.2007.10.004 18070628

